# SOX4 in chronic lymphocytic leukaemia: the forgotten transcription factor

**DOI:** 10.1007/s44313-025-00086-2

**Published:** 2025-07-04

**Authors:** Ricardo García-Muñoz, Jone Alberdi-Ballina, Giovanna Farfan-Quiroga, Eloy F. Robles, María José Larráyoz, María José Calasanz, José Ángel Martínez-Climent, Carlos Panizo, Javier Larreina-Pérez, Sofía Rincón-López, Johelys Atencio-Matos, Andrea Campeny-Najara, Ada Esteban-Figuerola, Montserrat Hernandez-Perez, Puy Garrastachu-Zumaran, María Velasco-Ruiz, Estefanía Ruiz de Gaona, Jesús Feliu

**Affiliations:** 1 Department of Hematology, Hospital Universitario San Pedro, La Rioja, 26006 Logroño, Spain; 2 Departamento of Hematology, Hospital Reina Sofía, Tudela, Navarra Spain; 3 Hemato-Oncology Program, Center for Applied Medical Research CIMA, University of Navarra, Idisna, Ciberonc, Pamplona, Spain; 4 CIMA-LAB Diagnostics-Clínica Universidad de Navarra, Navarra Institute for Health Research (IdiSNA, Pamplona, Spain; 5 Hospital Universitario Donostia, Biodonostia, Donostia, Spain; 6 Department of Diagnostic Imaging (Radiology) and Nuclear Medicine, University Hospital San Pedro and Centre for Biomedical Research of La Rioja (CIBIR), Logroño, La Rioja Spain; 7 Hematology and Hemotherapy Service, Fundación Hospital Calahorra, La Rioja, Spain

**Keywords:** Chronic Lymphocytic leukemia (CLL), SOX4 expression, IgHV mutational status, Cytogenetic Abnormalities, Prognostic Biomarkers, Risk Stratification

## Abstract

**Purpose:**

SRY-box transcription factor 4 (SOX4) is a transcription factor involved in early B cell development and has been implicated in various malignancies; however, its role in chronic lymphocytic leukemia (CLL) remains poorly understood. This study investigated the correlation between SOX4 expression and prognostic factors in CLL to determine its relevance to disease progression and clinical outcomes.

**Methods:**

A cohort of patients with CLL with a known immunoglobulin heavy chain variable region (IGHV) mutational status was analyzed for SOX4 expression using quantitative polymerase chain reaction (qPCR). Correlations between SOX4 levels and established prognostic markers including IGHV mutational status, cytogenetic abnormalities, and clinical outcomes were evaluated. Statistical analyses were performed to assess the association between SOX4 expression and patient survival.

**Results:**

Higher SOX4 expression was observed to be significantly associated with unmutated CLL (U-CLL) and adverse prognostic markers, including del(17)(p13). In contrast, lower SOX4 levels were observed in mutated CLL (M-CLL), and cytogenetic abnormalities were noted to be linked to favorable outcomes [del(13)(q21)]. Survival analysis indicated that elevated SOX4 expression was correlated with poor prognosis.

**Conclusion:**

SOX4 expression stratifies CLL subtypes and aligns with established prognostic markers. High SOX4 levels are associated with aggressive disease phenotypes, whereas low SOX4 expression is associated with better clinical outcomes. These findings indicate that SOX4 may serve as a potential biomarker for disease classification and risk stratification. Further studies are required to elucidate the biological significance of this phenomenon.

## Introduction

Chronic lymphocytic leukemia (CLL) is a malignancy characterized by clonal accumulation of mature-appearing CD5 + B cells [1]. Our research group previously explored the hypothesis that CLL cells originate from self-reactive B cells that have accumulated genetic damage and evaded normal immunological tolerance mechanisms, eventually transforming into malignant cells [2, 3]. In this context, CLL stem cells aberrantly generate an increased number of pro-B cells that possess an intrinsic propensity to develop into clonal CLL-like B cells with normal karyotype [4]. In addition, CLL B cells undergo continuous receptor editing [5] and exhibit “reversible anergy,” [6] two mechanisms that are closely linked to immunologic tolerance [7]. These altered immune tolerance processes may play a central role in shaping CLL development [8, 9]. Once lineage commitment is established, the composition of the B cell receptor (BCR) controls further development. Aberrant BCR signaling is associated with increased survival of malignant cells in CLL [10]. Several transcription factors have been implicated in CLL development [11]; however, the potential role of SRY-box transcription factor 4 (SOX4) in CLL pathogenesis remains poorly understood.

Notably, SOX4 plays a critical role in B cell development, as its absence results in developmental arrest during the pro-B to pre-B cell transition [12]. This blockade contrasts with observations in xenograft models of CLL, in which an abundant production of pro-B cells occurs that subsequently progress to monoclonal B-lymphocytosis, which is a precursor stage of CLL [4]. These findings indicate that although SOX4 deficiency impairs normal B cell maturation, the progression observed in CLL may be driven by antigen selection [4] or autonomous self-stimulation [10, 13], thus enabling the expansion of pro-B cells and their eventual malignant transformation [4]. Interestingly, a similar phenomenon has been observed in transgenic mice expressing catalytically inactive RAG1 (dominant-negative recombination activating gene 1 [dnRAG1] mice) [14, 15]. These mice develop early onset indolent CD5 + B-cell lymphocytosis due to a defect in secondary V(D)J rearrangements, which initiates the editing of autoreactive BCR specificity [14, 15]. Notably, SOX4 was overexpressed in these models [14], thereby reinforcing its potential role in the persistence and expansion of aberrant B cell populations.

Therefore, in the present study, we aimed to explore the correlation between SOX4 expression and the clinical or biological characteristics of patients with CLL.

## Patients and methods

### Data collection

Twenty-six unselected patients diagnosed with CLL according to the NCI-WG criteria [9] were included in this study. Peripheral blood samples obtained at diagnosis or before the first treatment were analyzed. This study was approved by the Institutional Review Board (IRB; approval number PI-176). The ethics committee responsible for overseeing this study is CEImLar, which ensures that the research complies rigorously with internationally recognized ethical frameworks, including the Nuremberg Code, Declaration of Helsinki, Belmont Report, and Oviedo Convention. The cells used in this study were obtained from the patients who provided informed consent for donation and use of blood and tissue samples for research purposes. In addition, the participants explicitly consented to the use of their anonymized data, thus ensuring the confidentiality and privacy of their personal information. Patient and disease characteristics are summarized in Table 1.

**Table 1:** Patient and disease characteristics

Characteristic	No.Patients	*Percentage*	Median (range)
Patient age, years			63 (34—81)
Patient sex, male/female	18/8	69%/31%	
CLL Binet stage			
A	14	54%	
B	8	31%	
C	4	15%	
Cytogenetics at diagnosis			
High risk^a^	15	54%	
Others	9	31%	
Null/not done	2	15%	
IGHV mutational status			
M-CLL	12	46%	
U-CLL	14	54%	
del(17)(p13.1)	3	13%	
del(11)(q22.3)	5	21%	
Trisomy 12	2	8%	
Normal FISH	8	33%	
del(13)(q14.3)	6	25%	
CLL treatment lines			
0	7	27%	
1	6	23%	
2	6	23%	
3 or more	7	27%	
Death during follow-up			
Yes	3	12%	
No	23	88%	

^a^del(17)(p13.1), del(11)(q22.3), U-CLL

*CLL* chronic lymphocytic leukemia, *IGHV* Immunoglobulin Heavy chain Variable, *M-CLL* CLL with mutated IGHV, *U-CLL* CLL with un-mutated IGHV

### Biological markers

Interphase fluorescence in situ hybridization (FISH) was performed to identify genomic aberrations and determine the mutation status of the rearranged immunoglobulin heavy chain (IGHV) as previously reported.

### Real-time quantitative reverse transcription-polymerase chain reaction (qRT-PCR)

Total RNA was extracted using TRIzol reagent (Invitrogen). cDNA synthesis was performed using M-MLV reverse transcriptase (Invitrogen), following previously reported methods. qPCR was performed in the 7300 PCR system (Applied Biosystems) using SYBR® Green PCR Master Mix (Applied Biosystems) to detect *SOX4* gene expression. The primers used in the study were *SOX4*-F, 5′-GGTCTCTAGTTCTTGCACGCTC-3′ and *SOX4*-R, 5′-CGGAATCGGCACTAAGGAG-3′. Data were normalized to *GAPDH* gene expression.

### Definitions

High *SOX4* expression was defined as a value > 1. Values ≤ 1 were considered low. The median *SOX4* level in the study population was used as the cutoff value.

IGHV with ≥ 98% sequence homology with the nearest germline V_H_ gene was defined as un-mutated CLL (U-CLL). IGHV with > 2% somatic mutations were considered mutated CLL (M-CLL).

High-risk patients with CLL were defined as those with U-CLL, del(17)(p13), and/or del(11)(q22.3).

### Statistical analysis

Descriptive statistics were used to process the patient and disease characteristics. Differences between proportions were assessed using the Chi-square test or Fisher’s exact test, whereas differences between means were evaluated using a two-tailed unpaired t-test or analysis of variance (ANOVA), followed by Scheffé’s multiple comparison test when applicable. Normality was assessed using the Shapiro–Wilk test, and non-Gaussian data were analyzed using the Kruskal–Wallis test, with the α-level adjusted for multiple comparisons using the Dunn-Sidak method. Overall survival (OS) was defined as the interval between diagnosis and date of censoring. The time to first treatment (TTFT) was defined as the interval from diagnosis to the initiation of therapy or the censoring date for untreated patients. Survival curves were created using the Kaplan–Meier method, and group comparisons were performed using log-rank or Breslow tests. Univariate and multivariate analyses of the risk factors associated with high SOX4 expression were conducted using logistic regression. The backward-elimination method was used to derive the final multivariate model. Statistical significance was set at p < 0.05.

## Results

**Fig. 1:**
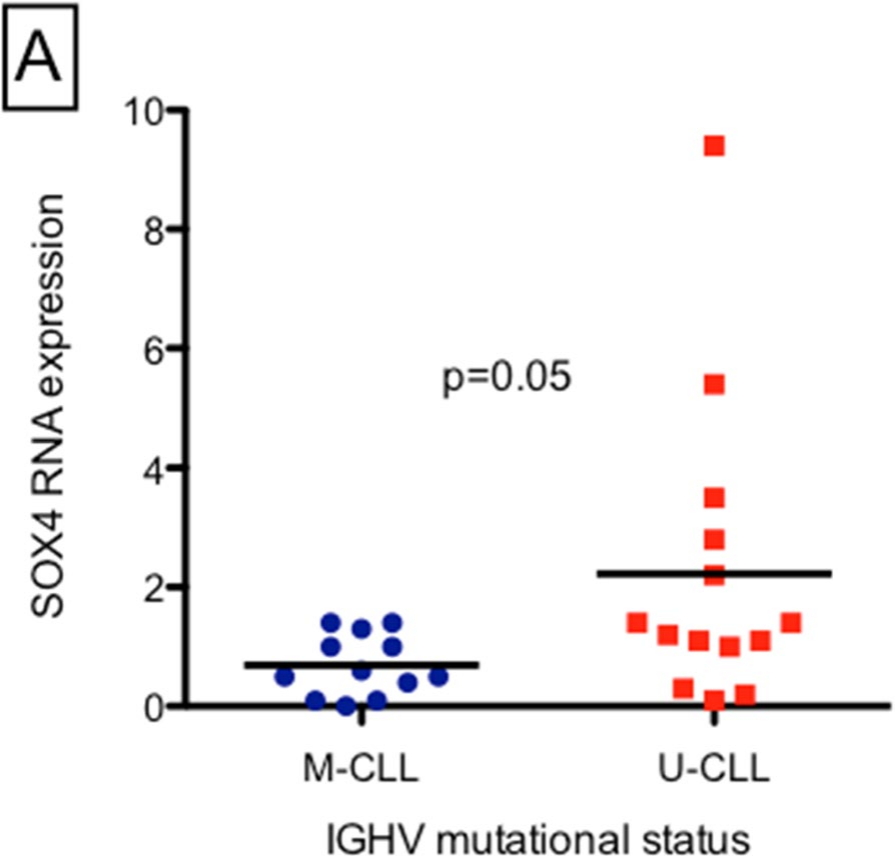
SOX4 gene expression according to the IGHV mutational status

We analyzed *SOX4* expression in 26 patients with CLL, with a median follow-up period of 71 months (range, 20–184 months). Mean gene expression of *SOX4* was 1.5 (range 0–9.4). Thirteen of the 26 (50%) patients had low *SOX4* expression, whereas the remaining 13 patients (50%) had high *SOX4* expression. The correlation of *SOX4* gene expression with IGHV mutational status (M-CLL vs. U-CLL) revealed that the U-CLL group expressed significantly higher levels of *SOX4* than the M-CLL group (2.2 vs. 0.7; *p* = 0.05) (Fig. 1). Therefore, high expression of *SOX4* is associated with U-CLL.

Of the 24 patients with available cytogenetic data, 8 (33%) had high-risk interphase cytogenetic abnormalities, and 16 (67%) had low/intermediate risk. Dunn-Sidak’s post hoc test demonstrated that patients with del(13)(q21) expressed lower levels of *SOX4* than those with del(17)(p13) (*p* = 0.04). The Kruskal–Wallis test revealed overall differences between individuals in *SOX4* expression (*p* = 0.02) and TTFT (*p* = 0.015) according to selected cytogenetic abnormalities. Patients with del(13)(q14) presented with a longer TTFT than those with del(11)(q23) (*p* < 0.0007).

Therefore, high gene expression of *SOX4* is associated with U-CLL IGHV mutational status and poor prognostic cytogenetic abnormalities del(17)(p13). In contrast, low levels of *SOX4* are associated with M-CLL and have good prognostic cytogenetic abnormalities, such as del(13)(q14).

Ten of the 13 (77%) patients with high *SOX4* expression required treatment during follow-up. The mean number of treatment lines administered was 2 (range 0–6), being 1 (range 0–3) in patients with low *SOX4* and 3 (range 0–6) in the high *SOX4* group. Median number of relapses in the high *SOX4* group was 2 (range 0–6), and this was 0.5 (range 0–2) in the low *SOX4* group. (Table 2. Summary of treatment requirements, treatment lines administered, and relapse statistics in patients with high and low SOX4.

**Table 2:** Summary of treatment requirements, treatment lines administered and relapse statistics in patients with high and low SOX4

Cytogenetics (FISH)/IGHV	SOX4 expression	*TTFT* *(months)*	Overall survival *(months)*	Treatment
	Mean 0.35 SD 0.24	Mean 46.96 SD 20.7	Mean 56,33 SD 23.17	
del(13)(q14.3)/M-CLL	0.0	69.08	69.08	
del(13)(q14.3)/M-CLL	0.6	64.49	64.49	
del(13)(q14.3)/M-CLL	0.5	43.28	43.28	
del(13)(q14.3)/M-CLL^a^	0.4	17.11	39.74	RB
del(13)(q14.3)/M-CLL	0.5	29.38	29.38	
del(13)(q14.3)/M-CLL^a^	0.1	58.43	92.16	R-FC
	Mean 2.05 SD 1.73	Mean 37.21 SD 27.61	Mean 104.21 SD 46.10	
Normal FISH/M-CLL^a^	1.0	83.18	183.77	C
Normal FISH/M-CLL^a^	1.0	24.49	83.54	R-HDMP, RB, O
Normal FISH/U-CLL	1.4	25.41	25.41	
Normal FISH/U-CLL ^b^	2.8	51.41	142.46	R-FMC, B, CHOP, A, R.HDMP
Normal FISH/U-CLL	1.2	25.44	114.33	
Normal FISH/U-CLL^a^	3.5	19.67	101.77	F, R-FC
Normal FISH/U –CLL^a^ Normal	0.1	67.61	90.00	R-FC
FISH/U –CLL +	5.4	0,46	92.43	F, R-CHOP, R-FCM
	Mean 0.56 SD 0.50	Mean 4.84 SD 6.87	Mean 69.71 SD 39.61	
del(11)(q22.3)/M-CLL^a^	0.1	2.98	47.67	RB, R-alk
del(11)(q22.3)/U-CLL^a^	1.1	1.21	118.13	C, R-CHOP, RB
del(11)(q22.3)/U-CLL^a^	1.1	16.95	100.52	R-FC, RB
del(11)(q22.3)/U-CLL^a^	0.3	2.79	61.9	R-HDMP, O
del(11)(q22.3)/U-CLL ^b^	0.2	0.26	20.33	R-HDMP
	Mean 4.33 SD 4.41	Mean 18.92 SD 21.93	Mean 71.00 SD 13.58	
del(17)(p13.1)/U-CLL^a^	2.2	13.57	86.66	R-FC, R.Hyer-CVAD, A, R-HDMP, BCR-inh
del(17)(p13.1)/U-CLL^a^	9.4	43.02	64.03	R-Hyper-CVAD/MA, A
del(17)(p13.1)/U-CLL^a^	1.4	0.16	62.33	R-CHOP, R-HDMP, RB, A, BCR-inh

^a^Patients that require treatment

^b^ Patients that death during follow-up

*M-CLL* CLL with mutated IGHV, *U-CLL* CLL with unmuated IGHV, *C *Chlorambucil, *R* Rituximab, *F* Fludarabine, *O* Obinutuzumab (GA-101), *A *Alemtuzumab, *R-FC *Rituximab fludarabine cyclophosphamide, *RB* Rituximab-Bendamustine, *R-FMC* Rituximab, fludarabine, mitoxantrone, cyclophosphamide, *R-HDMP* Rituximab high-dose methylprednisolone, *R-CHOP* Rituximab cyclophosphamide, doxorubicin, vincristine, prednisone, *R-Hyper-CVAD* hyper-fractionated cyclophosphamide, vincristine, doxorubicin and dexamethasone/methotrexate, cytarabine, *BCR-inh* B-cell receptor signalling inhibitors

**Fig. 2:**
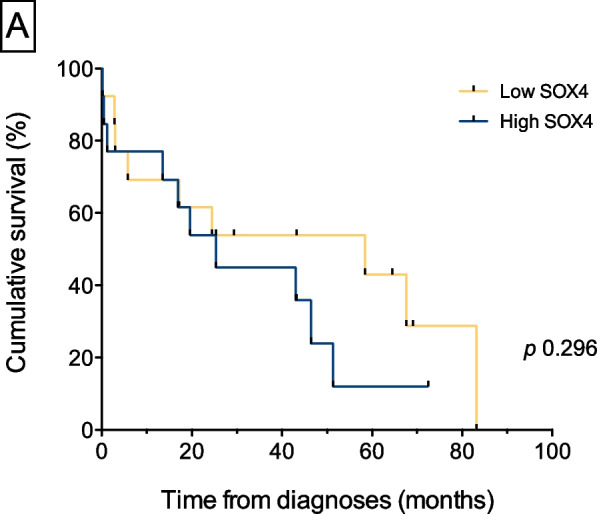
Analysis of TTFT survival with high and low SOX4 gene expression. The graph shows the mean TTFT and highlights differences in treatment initiation timing between the groups, despite absence of statistically significant differences

We analyzed TTFT and OS based on *SOX4* expression. The TTFT survival curves are shown in Fig. 2. (Analysis of TTFT survival with high and low SOX4 gene expression. The graph shows the mean TTFT and highlights differences in treatment initiation timing between the groups, despite absence of statistically significant differences). There were no statistically significant differences between the two groups in either TTFT (p = 0.296) or OS (*p* = 0.912). However, the mean TTFT was 36 months (range 0–83) in the low *SOX4* group and 28 months (range 0–73) in the high *SOX4* group. Although this difference was not statistically significant (probably due to the small sample size), patients with high *SOX4* levels required treatment approximately 8 months earlier than patients with low *SOX4*.

## Discussion

**Fig. 3:**
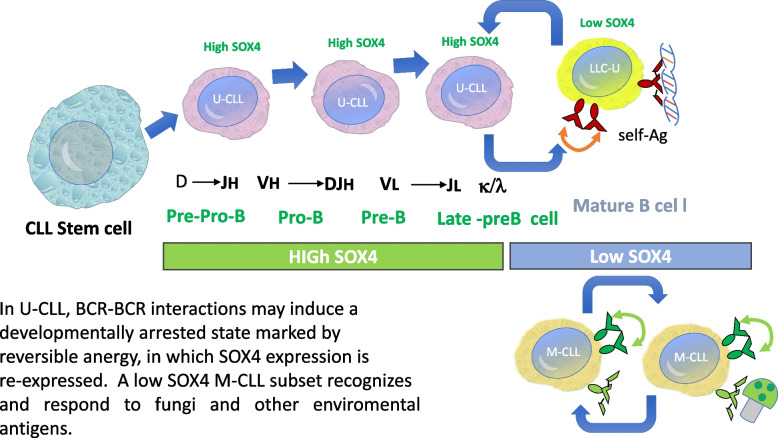
SOX4 levels and their potential role in CLL development

SOX4 is a key regulator of early B cell development, particularly during the transition from pre-pro-B to pro-B cells [16, 17]. In adult mice, SOX4 knockout did not affect mature B cells, supporting its specific role in early hematopoiesis [16, 17]. In the context of CLL, our findings indicate that SOX4 expression differs among the different disease subtypes. Low SOX4 expression in M-CLL is consistent with a more differentiated leukemic phenotype, potentially corresponding to memory B cells carrying IGHV mutations that recognize environmental antigens [18] or autoimmune-corrected cells [19, 20] that are regulated by immune tolerance mechanisms. Conversely, high SOX4 levels in U-CLL may reflect a less mature state, resembling xenograft models in which expanded pro-B cell populations [4] transition to pre-B cells under autonomous signaling interactions (BCR-BCR) [21–23]. This indicates that SOX4 may serve as a marker of immature CLL cells lacking IGHV mutations. (Fig. 3. SOX4 levels and their potential role in CLL development).

A similar phenomenon was observed in transgenic mice expressing catalytically inactive RAG1 (dnRAG1 mice), which developed early-onset indolent CD5 + B-cell lymphocytosis due to defective secondary V(D)J rearrangements required for BCR editing [14, 15]. Moreover, leukemic cells in these mice were predominantly unmutated and showed dysregulated SOX4 expression [14]. Interestingly, SOX4 was consistently overexpressed in these models [14], further supporting its potential role in maintaining aberrant B cell populations. This parallel relationship between the experimental models and CLL subtypes strengthens the hypothesis that SOX4 contributes to disease progression by influencing B cell differentiation.

Genomic aberrations play a well-established role in CLL prognosis [24]. Median survival estimates indicated that del(17p) and U-CLL were associated with a more aggressive clinical course, whereas M-CLL and del(13q21) were associated with significantly longer survival [24]. Therefore, we analyzed SOX4 expression in relation to known IGHV mutation status, revealing its association with poor prognosis subgroups, such as U-CLL and del(17p13), whereas low SOX4 levels correlated with favorable prognostic features, including M-CLL and del(13q21).

In addition to its role in early B cell differentiation, SOX4 is involved in regulating pre-B-cell survival and proliferation. Its gene product is essential for lambda5 expression, which is required for pre-BCR assembly, a stage preceding functional immunoglobulin rearrangements [25]. This aligns with its potential function in CLL stem cells, indicating that increased SOX4 expression may promote pro-B cell differentiation and support the proliferation and survival of self-reactive pre-B cells in the bone marrow.

Furthermore, transcriptional profiling across various lymphoma subtypes identified SOX4 as a molecular marker that is almost exclusively associated with CLL, reinforcing its specificity in disease biology. Although our sample size limited the survival analyses stratified by IGHV mutation status or cytogenetic abnormalities, our findings offer important insights into the prognostic implications of SOX4 expression in CLL.

Overall, these results indicate that SOX4 is an important factor in CLL progression. Future studies should focus on larger patient cohorts and functional assays to better define the biological roles and clinical significance of this disease.

## Conclusions

Our study shows the relevance of SOX4 expression as a molecular marker of CLL, particularly in distinguishing disease subtypes with different clinical outcomes. High SOX4 expression is associated with aggressive disease phenotypes, including U-CLL and del(17p13), both of which are associated with a poor prognosis. Low SOX4 expression was more commonly observed in the M-CLL and del(13)(q21) subgroups, which are typically characterized by indolent progression and a favorable prognosis.

Its near-exclusive association with CLL, as demonstrated by transcriptional profiling studies, further supports its potential as a biomarker for disease classification and risk stratification.

High SOX4 levels are associated with a more aggressive clinical course in U-CLL and del(17p13.1). In contrast, low SOX4 levels, which are associated with a more indolent disease course, have been frequently observed in M-CLL and del (13q21). Low SOX4 expression in M-CLL is consistent with a more differentiated leukemic phenotype carrying IGHV mutations that recognize environmental antigens (recognize and respond to fungi). In U-CLL, BCR-BCR interactions may induce a developmentally arrested state marked by reversible anergy, in which SOX4 is re-expressed.

## Data Availability

No datasets were generated or analysed during the current study.
